# ﻿Mitogenome and nuclear rRNA gene cluster of *Austropeplea subaquatilis* (Tate, 1880) from South Australia, with molecular and morphological comparison of A. cf. brazieri (Smith, 1882) from Victoria (Gastropoda, Hygrophila, Lymnaeidae)

**DOI:** 10.3897/zookeys.1255.164109

**Published:** 2025-10-09

**Authors:** Zhe-Yu Chen, Tanapan Sukee, Anson V. Koehler, Bonnie L. Webster, Robin B. Gasser, Winston F. Ponder, Neil D. Young

**Affiliations:** 1 Department of Veterinary Biosciences, Melbourne Veterinary School, Faculty of Science, The University of Melbourne, Parkville, VIC 3010, Australia The University of Melbourne Parkville Australia; 2 Department of Life Science, Natural History Museum, London, SW7 5BD, UK Natural History Museum London United Kingdom; 3 Australian Museum Research Institute, Australian Museum, Sydney, NSW 2010, Australia Australian Museum Sydney Australia

**Keywords:** Australia, freshwater snails, integrative taxonomy, mitochondrial genome, molecular phylogeny, nuclear rRNA gene cluster

## Abstract

Species of *Austropeplea* are lymnaeid snails endemic to Australia and New Zealand, and most are intermediate hosts of parasitic trematodes. Their taxonomy has long been uncertain due to the high phenotypic plasticity of most species. In this study, we used Oxford Nanopore sequencing technology to characterise the mitogenome and nuclear ribosomal RNA (rRNA) gene cluster of *Austropeplea subaquatilis* from South Australia to support comparative taxonomic investigations of this species. Then, *A. subaquatilis* was compared with A. cf. brazieri at both morphological and molecular levels. Morphologically, *A. subaquatilis* and A. cf. brazieri can be distinguished by shell morphometric indices, mantle edge morphology, pigmentation, and reproductive and neural anatomy. The two taxa differed by 1.9% in both the mitogenome and nuclear rRNA gene cluster. Sequence divergence was pronounced in the internal transcribed spacer (ITS) regions of the latter gene cluster, with nucleotide differences of 13.8% in ITS1 and 8.2% in ITS2. Phylogenetic analyses of sequence data for the mitochondrial *16S* gene and ITS2 placed the two taxa in distinct groups. Taken together, the integrative evidence presented herein supported species-level divergence between *A. subaquatilis* and A. cf. brazieri.

## ﻿Introduction

Lymnaeidae Rafinesque, 1815 (commonly known as pond snails) are a globally distributed group of freshwater hygrophilid snails, which have attracted widespread attention as intermediate hosts of various species of parasitic trematodes (e.g., Vinarski et al. 2019, 2023; Vázquez et al. 2023). The Australian native lymnaeid genus *Austropeplea* Cotton, 1942, is one of the vectors of *Fasciola hepatica* (liver fluke) disease in Australia and New Zealand (Boray and McMichael 1961; Boray 1964a, 1969, 1978; Ponder and Waterhouse 1997). Boray and McMichael (1961) synonymised all 23 previously named *Austropeplea* species to just one species, *Austropeplea tomentosa* (Pfeiffer, 1885), a name based on New Zealand specimens, although they recognised two distinct phenotypes A and B. The type A snails were darker with a smaller mantle border, and had a more robust and opaque shell with a distinct spire, while type B snails were lighter with a much larger mantle border, and with a fragile and transparent shell with a short spire (Boray and McMichael 1961; Boray 1964b). Despite the existence of two phenotypes, Boray and McMichael (1961) argued that they were all one species, based on assumed environment-mediated differences in snail phenotype and they cited evidence of successful hybridisation of the two phenotypes under laboratory conditions (Boray 1964b). In contrast, Puslednik et al. (2009), using morphological and molecular evidence, indicated that Australian *Austropeplea* represented a distinct lineage from the New Zealand *A. tomentosa*, and could be divided into different operational taxonomic units (OTUs). Based on these OTUs, Ponder et al. (2024) divided the Australian *Austropeplea* into four species in two subgenera, Austropeplea (Austropeplea) brazieri (Smith, 1882) which is widely distributed in eastern Australia, Austropeplea (Austropeplea) subaquatilis (Tate, 1880) in South Australia, and Austropeplea (Austropeplea) huonensis (Tenison Woods, 1876) and Austropeplea (Kutikina) hispida (Ponder & Waterhouse, 1997) in Tasmania. Nevertheless, due to limited defined morphological characters and the low phylogenetic resolution of current molecular markers, comprehensive species delimitation remains lacking for the nominal species other than A. (K.) hispida. As a result, the current classification of species (or OTUs) within the Australian members of typical *Austropeplea* largely relies on geographic origin rather than diagnostic features of the specimens themselves. Therefore, detailed morphological and molecular characterisation is needed to improve the taxonomic resolution within this genus.

The aim of this study was to characterise *A. subaquatilis* from South Australia morphologically and molecularly, and then compare this species with A. cf. brazieri from Victoria, Australia to establish their taxonomic and phylogenetic relationship.

## ﻿Materials and methods

### ﻿Sample collection and preservation

*Austropeplea subaquatilis* were collected from “Drain M” near Princes Highway in Thornlea, South Australia, Australia (latitude −37.36891397, longitude 140.2052126). Austropeplea cf. brazieri were previously collected from a roadside irrigation channel in Werribee South, Victoria, Australia (latitude −37.944706, longitude 144.698857) (see Sukee et al. 2024).

Both species of *Austropeplea* were then cultured in aquaria within a designated laboratory at The University of Melbourne, Victoria, Australia. Snails from different sources were maintained in strict isolation in separate tanks containing clean artificial pond water with aeration. Water was changed regularly and the snails were fed a commercial fish diet. A small section (~5 mm) of the foot muscle was excised from adult specimens and preserved in RNAlater at 4 °C for 24 h, -20 °C for one month and then stored at -80 °C until further processing. The remainder of each adult specimen was then placed in 70% ethanol for subsequent dissection and collection of morphological features.

### ﻿Gross morphology of mature snails

Animal external features in the living state were observed in the field and in individuals maintained in culture. Ten and 13 individuals of *A. subaquatilis* and A. cf. brazieri were dissected for internal morphological observation respectively, all individuals had attained a body size sufficient for oviposition. Terminology for the characters followed Ponder and Waterhouse (1997) and Vinarski and Pointier (2023). Images of living animals were captured using a Canon^®^ 5D Mark IV camera with a Canon^®^ EF 100 mm f/2.8L Macro IS USM lens. Preserved specimens were gently cleaned with soft brushes to remove the coagulated mucus and dissected under an Olympus SZ30 stereomicroscope. Detailed images of anatomical features were taken with the same Canon^®^ 5D Mark IV camera with a Laowa^®^ 25 mm f/2.8 2.5-5X Ultra Macro lens. The final high depth-of-field images were produced by a WeMacro^®^ Rail System and stacked from 20–30 single photos using Zerene Stacker^®^ 1.04. Anatomical illustrations were prepared using a Wild M5 stereomicroscope attached with a drawing tube. All images were modified and assembled using Adobe Photoshop 2023.

### ﻿Scanning electron microscopy

The radular sac was removed from three adult snails of each species and the soft tissue removed using a 10% potassium hydroxide (KOH) solution. After complete dissolution of soft tissues, each radula was washed extensively in MilliQ water. While still soft, each radula ribbon was transferred onto a round coverslip (Ø 13 mm), air-dried and fixed in place. The coverslip was then mounted onto an aluminium stub using conductive carbon tape (ProSciTech Pty Ltd). A 4-μm thick gold coating was deposited on the radula surface using SafeMatic^®^ CCU-010 coater. Radulae were then scanned using a Hitachi^®^ SU7000 scanning electron microscope under a low vacuum mode (5 kV). Images generated from the middle detector were used in this study.

### ﻿DNA isolation

Foot tissue of a specimen of *A. subaquatilis* (voucher number: AB291) was removed from the RNAlater and washed extensively in nuclease-free water (Qiagen). DNA was then isolated from each section of tissue using the E.Z.N.A. Mollusc DNA Kit according to manufacturer’s instructions (Omega Bio-tek Inc.). The quantity of DNA isolated from each tissue was determined using the Qubit 1X dsDNA HS Assay and a Qubit 2.0 Fluorometer 2 (Invitrogen, ThermoFisher).

### ﻿Target-enriched amplification of mitochondrial DNA

Amplification of the *A. subaquatilis* mitochondrial DNA was performed using the REPLI-g mitochondrial DNA kit (Qiagen) following the manufacturer’s protocol using a custom primer mix that was designed to match conserved regions of the lymnaeid *12S* and *16S* mitochondrial ribosomal subunits and the *cox*1 gene. Primers were 11–14 nt in length and incorporated phosphorothioate links between the last three bases at the 3’ end of the primers (Table 1). A total of 8 primers were combined into a 100 µM stock. Template DNA sample was diluted with water (supplied from the kit) to 150 ng in a total volume of 20 µL. A fresh amplification mix containing the custom primers was prepared as per manufacturer’s instructions (Qiagen). In brief, 29 µL of DNA template and primer mix was denatured at 75 °C for 5 min then cooled to room temperature. Next, 1 µL of REPLI-g Midi Polymerase was added, and the sample was incubated at 33 °C for 8 h. Finally, the polymerase was deactivated by raising the temperature to 65 °C for 3 min. Amplified DNA was quantified as described above and stored at 4 °C until further processing.

**Table 1.** Modified primers used to amplify lymnaeid mitochondrial DNA using the REPLI-g mitochondrial DNA amplification kit (Qiagen). Asterisks represent the incorporated phosphorothioate links.

Primer name	Primer sequence	Targeted region	Direction
RepGS_Aust16F	TACCTGTTTATC*A*A	*16S*	Forward
RepGS_16SBRis	AACTCAGATCAT*G*T	*16S*	Reverse
RepGS_12sF	CAACGGCAATAT*A*T	*12S*	Forward
RepGS_12sR	CTAGGATTAGAT*A*C	*12S*	Reverse
RepGT_JB3	ATCCT GAGGTTT*A*T	*cox*1	Forward
RepGT_JB4.5	ACATAATGAAAA*T*G	*cox*1	Reverse
RepGT_16sF	CCTTTTGCATCA*T*G	*16S*	Forward
RepGT_16sR	CGGTCTTAACTC*A*A	*16S*	Reverse

### ﻿Library preparation and long-read sequencing

For *A. subaquatilis*, a barcode was assigned to amplified DNA template using the RAPID 24 (SQK-RBK114) barcoding library kit (Oxford Nanopore Technologies) and loaded onto a R10.4.1 flow cell and sequenced for 4 h on the PromethION 2 Solo (Oxford Nanopore Technologies) sequencing platform. Post-sequencing base-calling of POD5 data was performed using the program Dorado v. 0.7.2 (Oxford Nanopore Technologies) in super-accurate mode and reads were stored in FASTQ format. Nanopore long read sequence data for A. cf. brazieri was available from a previous study (Sukee et al. 2024).

### ﻿Clustering analyses, mitochondrial genome assembly, and gene annotations

Long reads with homology to reference mitochondrial genomes or ribosomal RNA subunit of freshwater molluscs were identified using pblat v. 2.5.1 (Wang and Kong 2019) and extracted from the raw long read data file using seqtk v. 1.4-r122 (https://github.com/lh3/seqtk/). A consensus mitochondrial sequence or nuclear rRNA gene cluster sequence was assembled using canu v. 2.3 (https://github.com/marbl/canu) with minimum read length set to 1,000 bp and genome size set to 20,000 bp (mitochondrial genome) or 8,000 bp (nuclear rRNA gene cluster). The *A. subaquatilis* mitochondrial genome was perlimarly annotated using Geneious Prime v. 2024.0.7 and MITOS v. 2.0.2 (Bernt et al. 2013), then manully curated following the suggested roles for molluscs (Fourdrilis et al. 2018; Ghiselli et al. 2021). The published A. cf. brazieri mitochondrial genome (GenBank accession number PP100270) were also further curated according to the same criteria. All sequence annotations and GC content plots were visualised using the Proksee online server (Grant et al. 2023).

### ﻿Nucleotide diversity comparison

Nucleotide pairwise distances (p-distance) of complete mitochondrial genome, the nuclear rRNA gene cluster and each gene were calculated in Geneious Prime v. 2024.0.7 after alignment with Clustal Omega v. 1.2.3. Sliding window analyses of nucleotide diversity (300-bp windows with 10-bp steps for mitochondrial genome and 50-bp windows with 10-bp steps for nuclear rRNA gene cluster) were performed on the aligned mitogenomes and nuclear rRNA gene cluster of A. cf. brazieri and *A. subaquatilis* using the PopGenome package (Pfeifer et al. 2014) in R. For each comparison, nucleotide diversity values were plotted using the R package ggplot2 (Wickham 2016). The GC/AT content and skew values were calculated using a custom Python v. 3.9.13 script.

### ﻿Phylogenetic analyses

To compare A. cf. brazieri and *A. subaquatilis* samples with available molecular data for *Austropeplea* spp. (see Puslednik et al. 2009), phylogenies were reconstructed using only the mitochondrial *16S* and nuclear ITS2 regions (Suppl. material 1: table S1). *Orientogalba viridis* was used as outgroup (Liu et al. 2012; Suwancharoen et al. 2023). The *16S* and ITS2 sequences were aligned separately using MUSCLE v. 3.7 in AliView v. 1.28 and concatenated using catfasta2phyml (https://github.com/nylander/catfasta2phyml). Numbers of variable and parsimony informative sites of the nucleotide were calculated using MEGA v. 11.0.13. Partitioned Maximum Likelihood (ML) analyses with the majority-rule consensus tree were performed in IQ-TREE v. 1.6.12 (Minh et al. 2013) using Ultrafast fast bootstrap approach (Minh et al. 2013) with 10,000 reiterations. Bayesian inference (BI) was conducted in MrBayes v. 3.2.6 (Ronquist et al. 2012), with four independent runs, each of which was performed for 1,000,000 generations and sampled every 1000 generations with the first 25% samples discarded as burn-in. Convergence of the Markov chain Monte Carlo simulations was assessed to ensure that the average standard deviation of split frequencies was < 0.01 and the potential scale reduction factors (PSRFs) were ~1. Additionally, Tracer v. 1.7.2 (Rambaut et al. 2018) was used to verify that all effective sample size (ESS) values exceeded 200. The most appropriate model of sequence evolution (ML: K2P+G4 for ITS2 and K3Pu+F+G4 for *16S*; BI: K2P+G4 for ITS2 and HKY+F+G4 for *16S*) was selected using ModelFinder (Kalyaanamoorthy et al. 2017).

## ﻿Results

### ﻿Mitochondrial genome composition and comparison

**Figure 1.**
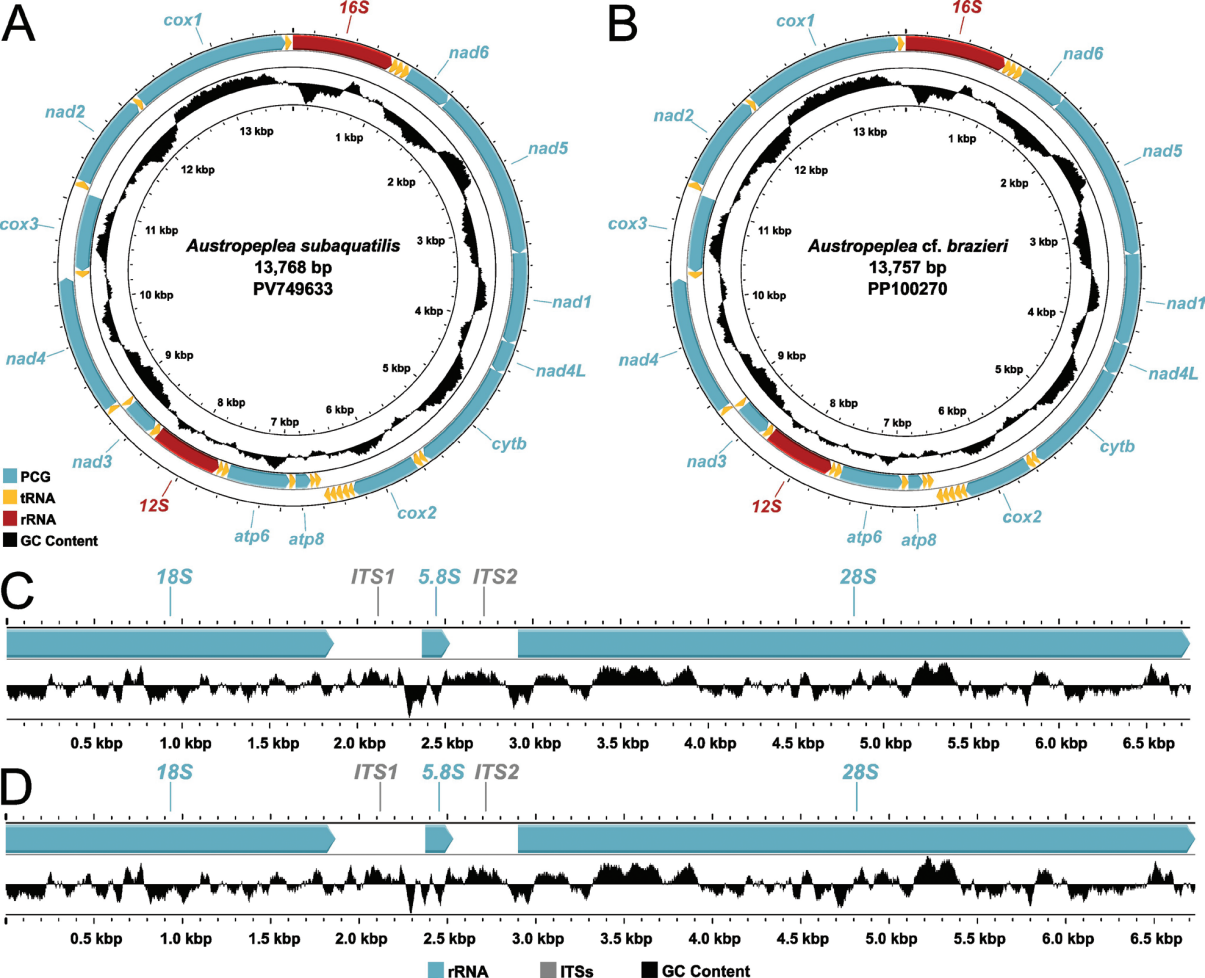
Reference mitochondrial genome and nuclear rRNA gene cluster of *subaquatilis* (A, C) and Austropeplea cf. brazieri (B, D). The direction of gene transcription is shown with an arrow. In each panel GC content is displayed via the observed skew patterns.

**Figure 2.**
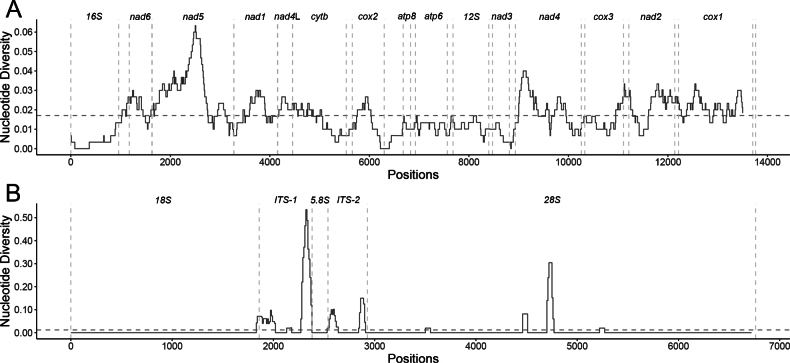
Sliding window analysis of the pairwise differences in the nucleotide identity of *Austropeplea subaquatilis* and Austropeplea cf. brazieri mitochondrial genomes (A) and nuclear rRNA gene cluster (B). Gene boundaries are indicated by vertical dotted lines. The horizontal dotted line indicates the average nucleotide diversity between the two sequences.

The mitochondrial genomes (mitogenome) of *A. subaquatilis* (GenBank accession number PV749633) and A. cf. brazieri (GenBank accession number PP100270) are 13,768 and 13,757 bp, respectively (Fig. 1A, B). Both genomes contain 37 genes: 13 protein-coding genes (PCGs), 2 ribosomal RNA genes (rRNAs), and 22 transfer RNA genes (tRNAs) (Fig. 1A, B, Table 2). Eight (*nad*4L, *cytb*, *cox*1, *cox*2, *atp*8, *atp*6, *nad*3, *nad*2) of the PCGs terminate with a truncated stop codon, which is assumed to be completed as TAA by the addition of 3’ A residues to the mRNA during transcription. The arrangement of genes within both mitogenomes are identical with only minor differences in inferred lengths of non-coding tRNA (tRNA-D, F, W, C, G, H, Q, L2, M, T and K) and *12S* genes (Table 2). The mitogenome nucleotide composition of *A. subaquatilis* is 32.8% A, 40.7% T, 12.4% C and 14.1% G, resulting in an AT content of 73.4%, which is nearly same as the 73.3% observed in A. cf. brazieri (with 32.8% A, 40.5% T, 12.5% C and 14.2% G). The AT/GC skew values for *A. subaquatilis* and A. cf. brazieri are -0.1072/0.0624 and -0.1051/0.0612, respectively. The overall plots of GC content in *A. subaquatilis* closely resembles those observed in A. cf. brazieri, with no substantial differences in content or distribution patterns across the mitogenomes (Fig. 1A, B). Both species exhibit higher GC content in the *cox3* and *cox*1 regions and reduced GC content in *nad*2, *nad*6, and *16S*rRNA. The overall mitochondrial genome p-distance between *A. subaquatilis* and A. cf. brazieri is 1.9%. The p-distance of each of the PCGs range from 3.1% (*nad*5) to 0.6% (*16S*) (Fig. 2A, Table 2).

**Table 2.** Location, lengths, and directions of annotated genes within the mitochondrial and nuclear rRNA gene clusters of *Austropeplea subaquatilis*, with comparisons to Austropeplea cf. brazieri (values after slash). Nucleotide pairwise identity of each gene between the two species is shown.

Gene designations	Location start	Location end	Length (bp)	Direction	Pairwise Identity	p-distance
Mitochondrial genes						
*16S*	1/1	986/986	986/986	forward	99.4%	0.6%
tRNA-L1(tag)	988/988	1051/1051	64/64	forward	N/A	N/A
tRNA-P(tgg)	1047/1047	1106/1106	60/60	forward	N/A	N/A
tRNA-A(tgc)	1107/1107	1170/1170	64/64	forward	N/A	N/A
*nad*6	1171/1171	1629/1629	459/459	forward	97.6%	2.4%
*nad*5	1631/1631	3277/3277	1647/1647	forward	96.9%	3.1%
*nad*1	3279/3279	4154/4154	876/876	forward	98.3%	1.7%
*nad*4L	4155/4155	4452/4452	298/298	forward	98.3%	1.7%
*cytb*	4453/4453	5533/5533	1081/1081	forward	98.3%	1.7%
tRNA-D(gtc)	5536/5536	5588/5587	53/52	forward	N/A	N/A
tRNA-F(gaa)	5589/5588	5651/5650	63/63	forward	N/A	N/A
*cox*2	5652/5651	6294/6293	643/643	forward	98.3%	1.7%
tRNA-Y(gta)	6297/6296	6346/6345	50/50	forward	N/A	N/A
tRNA-W(tca)	6347/6346	6405/6405	59/60	forward	N/A	N/A
tRNA-C(gca)	6410/6410	6468/6468	59/59	forward	N/A	N/A
tRNA-G(tcc)	6471/6470	6524/6522	54/53	forward	N/A	N/A
tRNA-H(gtg)	6527/6525	6583/6582	57/58	forward	N/A	N/A
tRNA-Q(ttg)	6592/6591	6650/6649	59/59	reverse	N/A	N/A
tRNA-L2(taa)	6651/6650	6703/6701	53/52	reverse	N/A	N/A
*atp*8	6705/6703	6855/6853	151/151	reverse	98.2%	1.8%
tRNA-N(gtt)	6857/6854	6920/6917	64/64	reverse	N/A	N/A
*atp*6	6921/6918	7560/7557	640/640	reverse	98.6%	1.4%
tRNA-R(tcg)	7561/7558	7623/7620	63/63	reverse	N/A	N/A
tRNA-E(gaa)	7624/7621	7675/7672	52/52	reverse	N/A	N/A
*12S*	7676/7673	8393/8388	718/716	reverse	98.2%	1.8%
tRNA-M(cat)	8394/8389	8466/8457	73/69	reverse	N/A	N/A
*nad*3	8467/8458	8806/8797	340/340	reverse	99.1%	0.9%
tRNA-S2(tga)	8817/8808	8871/8862	55/55	reverse	N/A	N/A
tRNA-S1(gct)	8872/8863	8926/8917	55/55	reverse	N/A	N/A
*nad*4	8927/8918	10252/10243	1326/1326	forward	98%	2%
tRNA-T(tgt)	10253/10244	10319/10311	67/68	reverse	N/A	N/A
*cox*3	10321/10313	11100/11092	780/780	reverse	98.8%	1.2%
tRNA-I(gat)	11141/11133	11205/11197	65/65	forward	N/A	N/A
*nad*2	11206/11198	12109/12101	904/904	forward	97.8%	2.2%
tRNA-K(ttt)	12110/12102	12191/12180	82/79	forward	N/A	N/A
*cox*1	12203/12192	13694/13683	1492/1492	forward	97.7%	2.3%
tRNA-V(tac)	13695/13684	13755/13744	61/61	forward	N/A	N/A
Nuclear rRNA gene cluster						
*18S*	1/1	1865/1865	1865/1865	forward	100%	0%
ITS1	1866/1866	2374/2369	509/504	forward	86.2%	13.8%
*5.8S*	2375/2370	2532/2527	158/158	forward	100%	0%
ITS2	2533/2528	2900/2915	368/388	forward	91.8%	8.2%
*28S*	2901/2916	6731/6747	3831/3832	forward	99.3%	0.7%

### ﻿Nuclear rRNA gene cluster composition and comparison

The completed nuclear rRNA gene cluster of *A. subaquatilis* (GenBank accession no. PV593739) and A. cf. brazieri (GenBank accession no. PV593740) span 6,712 bp and 6,747 bp respectively (Fig. 1C, D). The two ribosomal DNA sequences exhibit similar overall structures, comprising the *18S*rRNA, *5.8S*rRNA, *28S*rRNA genes and two internal transcribed spacer regions (ITS1 and ITS2) (Fig. 1C, D, Table 2). Both sequences share identical lengths for the *5.8S*rRNA (158 bp). The *18S*rRNA and *28S*rRNA regions measure 1,865 bp and 3,815 bp in *A. subaquatilis*, and 1,866 bp and 3,832 in A. cf. brazieri, respectively. Notable differences are observed in the ITS regions: *A. subaquatilis* contained a 509 bp ITS1 and a 368 bp ITS2, whereas A. cf. brazieri possesses a 504 bp ITS1 and a 388 bp ITS2 (Table 2). The nuclear rRNA gene cluster of *A. subaquatilis* and A. cf. brazieri show similar nucleotide compositions: 22.8% A, 21.8% T, 25.7% C, and 29.7% G in *A. subaquatilis*; 22.5% A, 22.0% T, 25.7% C, and 29.8% G in A. cf. brazieri. Both sequences have consistent GC content (55.4% and 55.5%, respectively) and similar GC skew values, while *A. subaquatilis* shows slightly higher AT skew than A. cf. brazieri (AT/GC skew: 0.0227/0.0715 and 0.0123/0.0737). The GC content plots of the nuclear rRNA gene cluster in *Austropeplea subaquatilis* and A. cf. brazieri are similar (Fig. 1C, D). In both species, the GC content plots exhibit several local maxima in GC proportion within the central region of the *28S*rRNA gene, while pronounced decreases in GC proportion were observed at the boundaries of the internal transcribed spacers (ITS1 and ITS2), particularly at their junctions with the adjacent *5.8S* and *28S*rRNA genes. The overall nuclear rRNA gene cluster p-distance between *A. subaquatilis* and A. cf. brazieri was 1.9%. While the rRNA genes are largely identical (99%–100%), the ITS regions exhibit substantial sequence divergence, with the p-distance of ITS1 and ITS2 regions being 13.8% and 8.2%, respectively (Fig. 2B, Table 2).

### ﻿Phylogenetic analysis

**Figure 3.**
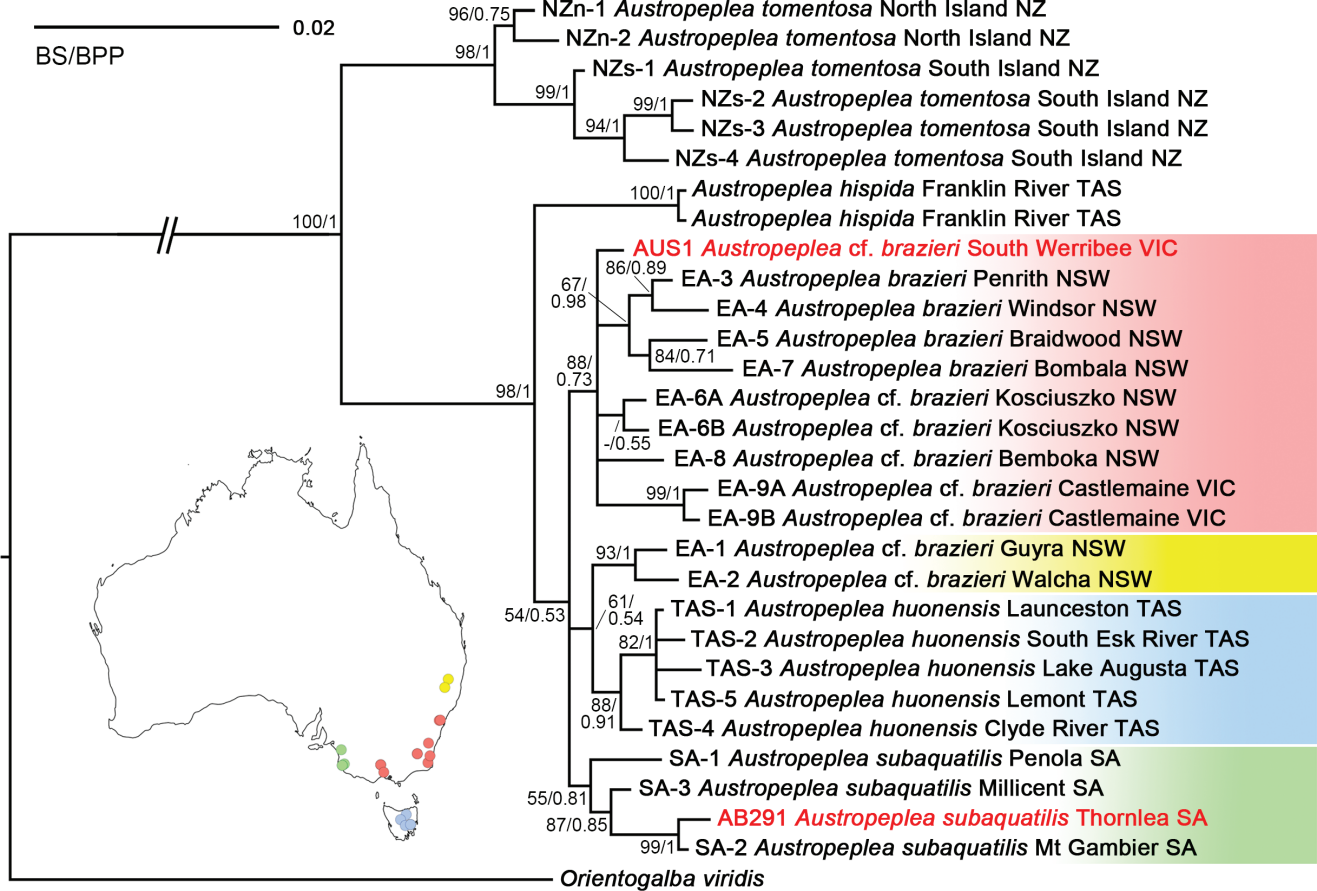
Bayesian phylogenetic tree of *Austropeplea* based on combined *16S* and ITS2 sequences. Red tip labels represent the sequences generated in this study. Coloured shading and the corresponding inset map summarise the geographic distributions of the principal lineages. Maximum likelihood (ML) bootstrap (BS) and Bayesian posterior probability (BPP) support values for shown for each node. Scale bar represents substitutions per site.

Phylogenetic trees were constructed from a dataset consisting of 29 *16S* + ITS2 sequences of *Austropeplea*, 27 of them were obtained from previous study (Puslednik et al. 2009), two were generated for this study and one available outgroup taxon (Liu et al. 2012; Suwancharoen et al. 2023). The aligned lengths of *16S* and ITS2 genes were 433 and 452 nucleotides, respectively. Within these sequences, 79 and 87 sites were variable, while 69 and 46 sites were parsimony informative. The Bayesian-inferred (BI) and maximum likelihood (ML) phylogenetic trees of *Austropeplea* were not fully resolved. Nevertheless, both Bayesian posterior probabilities (BPP) and ML bootstrap (BS) values supported separation between Australian and New Zealand species. *Austropeplea hispida* was recovered as sister to the remaining Australian species, which together formed a trichotomy in the inferred topology (Fig. 3). The sequences generated here for *Austropeplea subaquatilis* and A. cf. brazieri group with the southern Australian and eastern Australian samples from previous study, respectively.

### ﻿Taxonomic account

#### ﻿ Austropeplea subaquatilis

Taxon classificationAnimaliaLymnaeidaLymnaeidae

(Tate, 1880)

139D207B-88EA-53CF-A06B-7008B9EDCB8D

**Figure 4.**
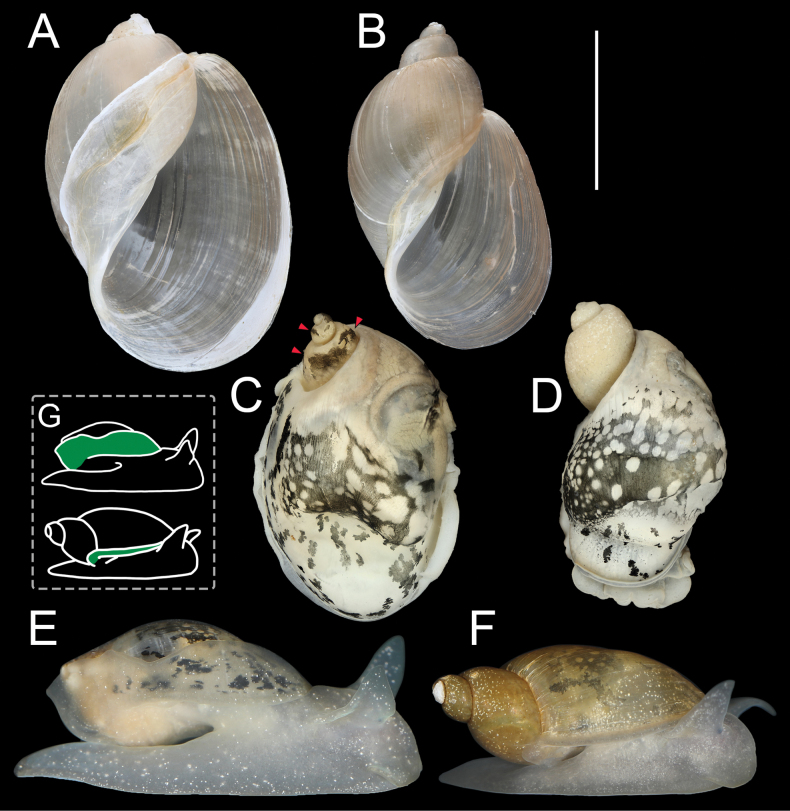
Shells (A, B), mantle pigmentations (C, D) and living individuals (E, F) of *Austropeplea* spp. A, C, E. *Austropeplea subaquatilis*; B, D, F. Austropeplea cf. brazieri. G. Schematic representation of mantle extension ranges (green areas), *A. subaquatilis* shown above, A. cf. brazieri below. The red arrows (in C) indicate the mantle pigmentation on the visceral coil. Scale bar: 5 mm (except G not to scale).

**Figure 5.**
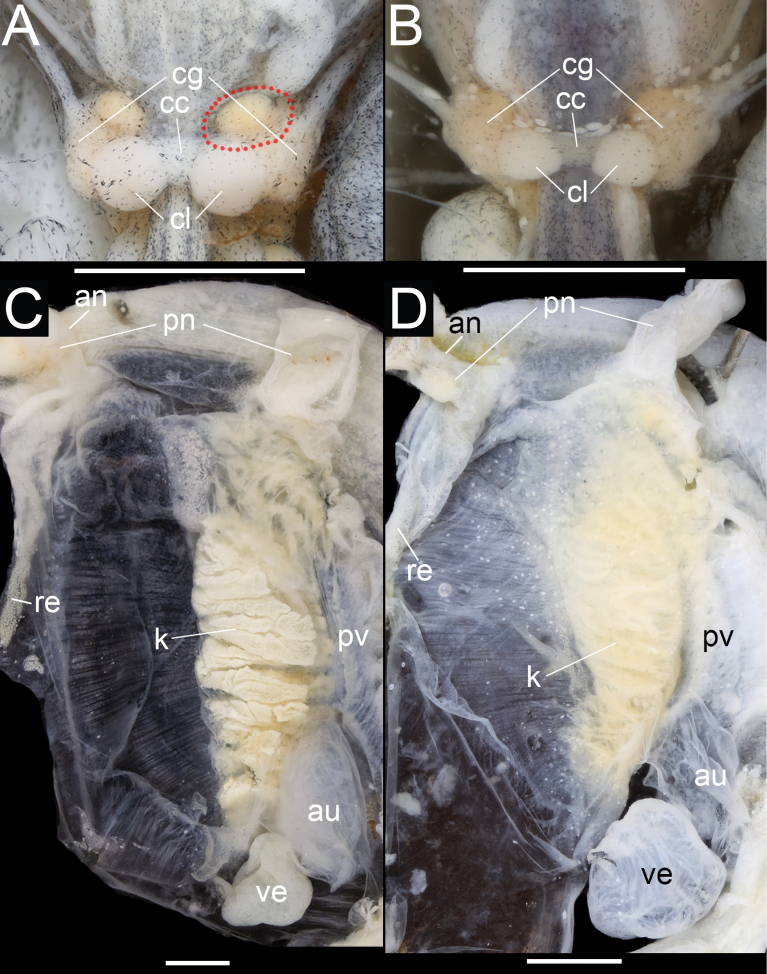
Dorsal side of central nervous system (A, B) and pallial complex (C, D) of *Austropeplea* spp. A, C. *Austropeplea subaquatilis*; B, D. Austropeplea cf. brazieri. Abbreviations: an – anus, au – auricle, cc – cerebral commissure, cg – cerebral ganglia, cl – commissural lobule, k – kidney, pn – cut wall of pneumostome, pv – pulmonary vein, re – rectum, ve – ventricle. The red-dotted line shows the additional lobe from the cerebral ganglion with a clear boundary. Scale bars: 1 mm.

**Figure 6.**
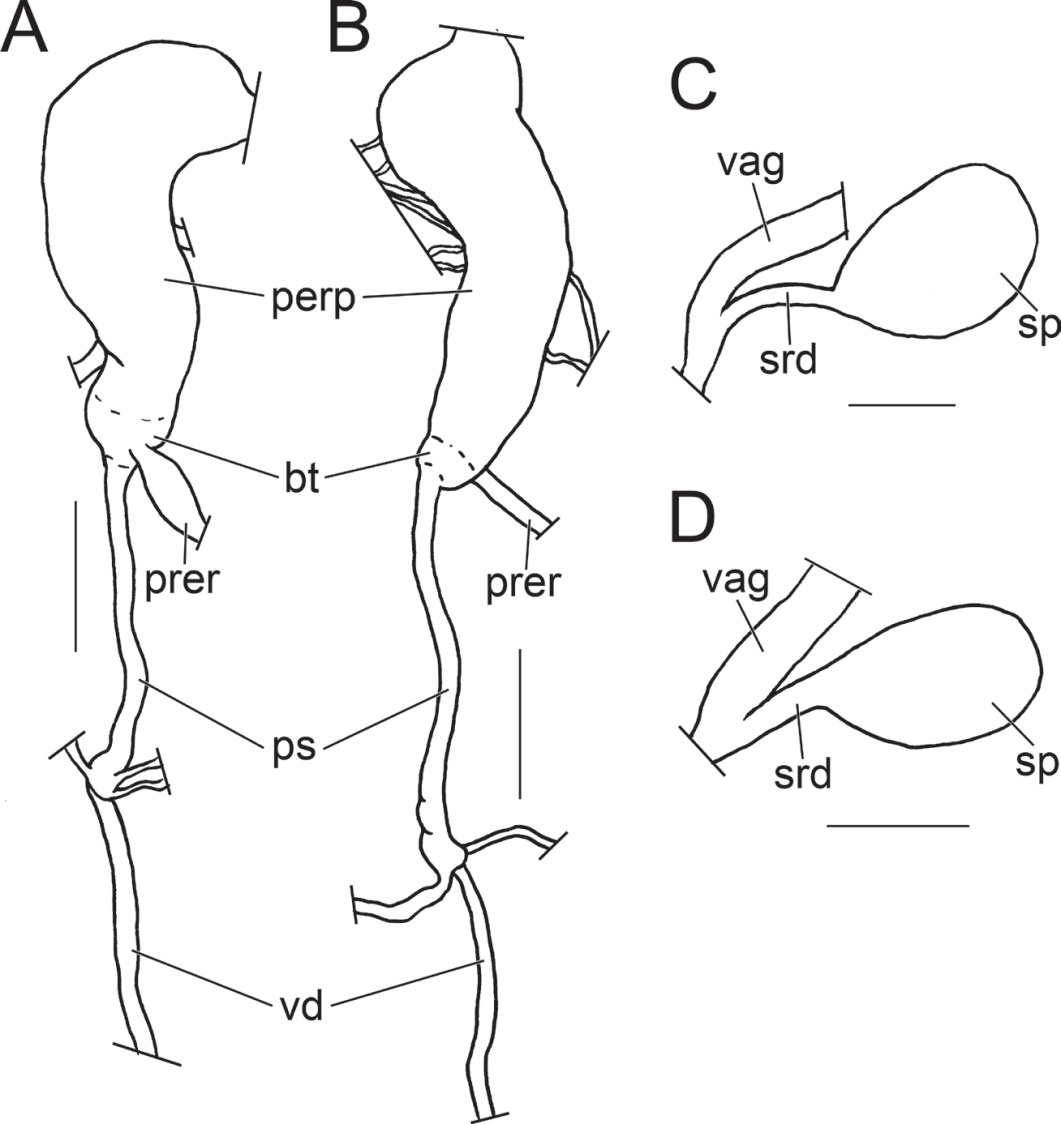
Male (A, B) and female (C, D) copulatory apparatus of *Austropeplea* spp. A, C. *Austropeplea subaquatilis*; B, D. Austropeplea cf. brazieri. Abbreviations: bt – bulbous termination of praeputium, prep – praeputium, prer – praeputium retractor muscles, ps – penis sheath, sp – spermatheca, srd – spermatheca duct, vag – vagina, vd – vas deferens. Scale bars: 1 mm.

**Figure 7.**
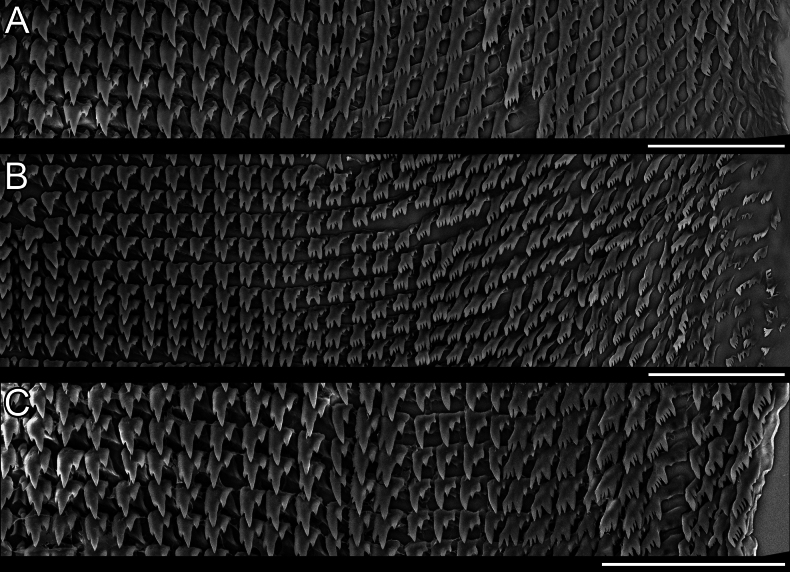
Radulae of *Austropeplea* spp. A. *Austropeplea subaquatilis*; B. Normal form of Austropeplea cf. brazieri; C. Variant form of Austropeplea cf. brazieri. Scale bars: 100 μm.

Figs 4A, C, E, 5A, C, 6A, C, 7A

##### Material examined.

Specimens from “Drain M” near Princes Highway in Thornlea, South Australia, Australia, and their artificially bred offspring.

##### Description.

***Shell*** (Fig. 4A) medium in size (up to 12.5 mm in height), ovate, with low, narrow conical spire and strongly inflated last whorl. Shell wall thin, fragile in some specimens. Whorls (4.0–4.5 in number) rounded, slightly convex, separated by a shallow, slightly oblique to nearly straight suture. Last whorl comprises ~0.9 of shell height. Shell surface smooth, somewhat shiny, light brown to nearly colourless, covered by collabral growth lines. Aperture pyriform, with evenly rounded basal and palatal margins, posterior corner forming angle with last whorl. Peristome sharp, not expanded but columellar lip reflexed and attached to back of last whorl. Parietal callus thin but distinct, extending to last whorl far beyond inner lip. Columellar fold weakly developed. Umbilicus covered by inner lip, closed or very narrow (slot-like).

***Head-foot*** (Fig. 4C, E) typical of family. Foot broad, reaching 1.5–2× shell height when fully extended, light grey with sparse white freckles (observed when living). When stimulated, considerable quantities of mucus produced and covers entire body. Tentacles shield-shaped (Fig. 4E), twice as long as wide (observed when living). Mantle light grey with large black blotches on pallial roof. Mantle collar (Fig. 4E) reflexed and attached to shell, extended as thin flap on both sides to enclose shell fully or largely in mature individuals. Closed edge of mantle collar situated along midline of animal, forming marginal fold near shell apex. Mantle covering visceral coil with band-like black pigment in mature individuals (Fig. 4C); disconnected from pigmentation on pallial roof, and readily lost in preserved specimens.

***Central nervous system*** typical of family (Fig. 5A). Cerebral ganglia with regular borders, pale yellow (fresh). Commissural lobule distinct, white, approximately equal to cerebral ganglia in size.

***Pulmonary roof*** (pallial complex) (Fig. 2C) with heart and kidney in their typical positions for family. Kidney spindle-shaped, thin-walled, with transversely pleated lining of sinuate tube visible through transparent wall, proximal part opposite anterior pericardium. Ureter short, urinary opening not observed.

***Prostate*** pear-shaped, with single internal fold. Sperm duct long and thick, equal to or slightly longer than oothecal gland in length. Praeputium (Fig. 6A) light greyish-white, cylindrical, tapers towards proximal end, distal part folded near male genital opening. Bulbous termination of praeputium distinct in lighter colour to white, equal to or wider than narrowest part of praeputium. Penis sheath narrow, shorter than praeputium, proximal part slightly inflated. ICA 1.34 to 2.02. Spermatheca (Fig. 6C) spherical, duct short, not exceeding length of spermatheca, width approx. 0.1 of its length.

***Radula*** of the haplolateral multidentate type (Fig. 7A). Radular formula 28-C-28 to 32-C-32. Teeth in same row bend upward to margin. Central tooth small, bicuspid, asymmetrical, right cusp significantly larger than left. Lateral teeth pairs 1–8~11 tricuspid, middle cusp largest, left cusp larger than right, rarely with denticle situated on the right basal side; pairs 9~12–28~32 with four or five cusps.

##### Remarks.

The name *A. subaquatilis* (Tate, 1880: 103, pl. 4, Fig. 5A–C) is here tentatively considered to be the species name for South Australian *Austropeplea* populations following Ponder et al. (2024). This species was described based on type material collected from the River Torrens in Adelaide and are not aware of it having been found from type locality in recent years (Z.-Y. Chen, unpublished data). A currently recognised synonym, *A. aruntalis* Cotton & Godfrey, 1938 (replacement name for *Limnaea papyracea* Tate, 1880: 103, pl. 4, Fig. 6A, B), may represent a valid species name for the South Australian Limestone Coast population, from which the specimens in this study were collected, if future studies reveal consistent differences between populations of the two forms.

##### Distribution.

South-eastern South Australia and western Victoria (Ponder et al. 2024).

#### ﻿ Austropeplea cf. brazieri

Taxon classificationAnimaliaLymnaeidaLymnaeidae

(E. A. Smith, 1882)

1ABD9CFF-7113-5680-B5EB-D52B16ACC5BB

Figs 4B, D, F, 5B, D, 6B, D, 7B

##### Material examined.

Artificially bred specimens in the lab, which were originally from Werribee South, Victoria, Australia.

##### Description.

***Shell*** (Fig. 4B) medium in size (≤14.7 mm in height in lab condition), high-conical, with relatively narrow and high spire and moderately inflated body whorl. Shell thin but somewhat solid, in some specimens almost translucent. Whorls (4–4.5 in number) rounded, convex, slowly increasing, separated by deep and oblique suture. Shell surface smooth, light brown, covered by collabral growth lines. Aperture pyriform, with evenly rounded basal and palatal margins. Peristome sharp, not expanded but columellar lip slightly reflexed. Parietal callus thin but distinct, extending a little over the parietal wall. Columellar fold weakly developed. Umbilicus covered by inner lip, closed or very narrow (slot-like).

***Head-foot*** (Fig. 4D, F) typical of family. Foot broad, fully extended approximately equal to shell height, light grey with dense white freckles (observed when living). Tentacle elongated triangular, length equal to width (observed when living). Mantle light grey with large black blotches on pallial roof. Mantle collar slightly reflexed and attached to aperture margin (Fig. 4F). Mantle covering visceral coil unpigmented (Fig. 4D).

***Central nervous system*** (Fig. 5B) typical of family. Cerebral ganglia with irregular-borders, pale yellow in fresh material. Commissural lobule distinct, white, notably smaller in size than cerebral ganglia.

***Pulmonary roof*** (Fig. 5D) with heart and kidney in typical positions for family. Kidney spindle-shaped, thin-walled, with transversely pleated lining of sinuate tube visible through translucent wall, proximal part opposite to anterior pericardium. Ureter short, urinary opening not observed.

***Prostate*** fusiform, with single internal fold. Sperm duct short, almost invisible in natural position. Praeputium (Fig. 6B) light greyish white, cylindrical, tapers towards proximal end, distal part near opening folded. Bulbous termination of praeputium distinct in lighter colour to white, narrowest across praeputium. Penis sheath narrow, shorter than praeputium, proximal part slightly inflated. Index of copulatory apparatus (ICA) 1.12 to 2.21. Spermatheca (Fig. 6D) ellipsoid. Spermatheca duct shorter than length of spermatheca, width approx. quarter of length, proximal external side somewhat adherent to the vaginal duct.

***Radula*** of the haplolateral multidentate type (Fig. 7B). Radular formula 28-C-28 to 38-C-38. Teeth in same row approximately aligned horizontally. Central tooth small, bicuspid, asymmetrical, right cusp significantly larger than left. Lateral teeth pairs 1–11~13 tricuspid, middle cusp largest, left cusp larger than right, rarely with denticle situated on the right basal side; pairs 12~14–28~38 with four or five cusps. A variant individual observed among examined specimens (Fig. 7C), with lateral teeth pairs 1–13 tricuspid, middle cusp largest, left cusp larger than right, rarely left cusp absent; pairs 14–17 tricuspid, left cusp largest, right two cusps gradually reduced; pairs 18–28 with 3–6 cusps.

##### Remarks.

We use the species-group name A. cf. brazieri for our Victorian specimens because both molecular and morphological data indicate clear differences from typical *Austropeplea brazieri* (E. A. Smith, 1882: 274, pl. 5, fig. 15, from Glebe Point, Sydney, New South Wales). The currently recognised distribution of *A. brazieri* is broad (Ponder et al. 2024), and our analysis and that done previously by Puslednik et al. (2009) have shown that it does not form a monophyletic group. To reflect this taxonomic uncertainty, we follow our earlier usage (Sukee et al. 2024) in applying the provisional name A. cf. brazieri to the Victorian population.

There are three additional names available for this taxon, all from New South Wales (*Glacilimnaea gelida* Iredale, 1943: 214, Blue Lake, Mt Kosciusko, NSW; *Simlimnea morbida* Iredale, 1944: 119, figs 5-4, Walcha, NSW; and *Simlimnea aegrifer* Iredale, 1944: 119, fig. 5-5, Bombala, NSW), but based on the molecular data of species from representative location of above (Fig. 3), none are applicable to the Victorian taxon.

##### Distribution.

Victoria, Australia.

### ﻿Morphological remarks

Due to its short apex and very large aperture, *A. subaquatilis* can be distinguished from A. cf. brazieri by shell morphometrics such as the Index Spire height/Shell height and Index Aperture height/Shell height (Table 3). A distinguishing feature that allows mature individuals of *A. subaquatilis* to be readily separated from those of A. cf. brazieri is the markedly expanded and extended mantle edge in *A. subaquatilis*. Additionally, the shell of *A. subaquatilis* is fragile and relatively small compared to the broad foot of the animal. Under conditions of high population density and limited food availability in aquaria, *A. subaquatilis* often entered a state of developmental arrest (diapause), producing dwarf forms lacking mantle extension, yet remaining reproductively functional. When these dwarf individuals were transferred to low-density environments with adequate food supply, they resumed growth and eventually developed an expanded mantle that enclosed the shell, similar to wild-type adults. This phenomenon has not been observed in A. cf. brazieri under same laboratory conditions.

**Table 3.** Morphometric characteristics of studied specimens of *Austropeplea subaquatilis* and Austropeplea cf. brazieri. ICA: Index of the copulatory apparatus, ratio of praeputium length and penis sheath length. Parenthetical values represent the mean of the group, * indicates p < 0.001.

Characters and Indexes	Species		p
	*A. subaquatilis* (*n* = 10)	A. cf. brazieri (*n* = 13)	
Number of whorls	3.75–4.5	4–4.25	
Shell height (SH), mm	9.02 – (10.37) – 12.5	9.71 – (11.30) – 14	
Shell width (SW), mm	6.04 – (6.87) – 8.41	5.92 – (7.14) – 8.98	
Spire height (SpH), mm	1.45 – (2.07) – 2.63	2.86 – (3.79) – 4.66	*
Body whorl height (BWH), mm	8.58 – (9.54) – 11.45	8.63 – (9.95) – 12.19	
Aperture height (AH), mm	7.27 – (8.20) – 9.75	6.36 – (7.46) – 9.5	
Aperture width (AW), mm	4.29 – (5.05) – 6.48	3.84 – (4.72) – 6.12	
Index SW/SH	0.63 – (0.66) – 0.70	0.55 – (0.63) – 0.69	
Index SpH/SH	0.16 – (0.20) – 0.21	0.28 – (0.34) – 0.41	*
Index BWH/SH	0.87 – (0.92) – 0.95	0.86 – (0.88) – 0.92	
Index AH/SH	0.75– (0.79) – 0.85	0.59 – (0.66) – 0.71	*
Index AW/AH	0.44 – (0.49) – 0.52	0.34– (0.42) – 0.45	*
ICA	1.34 – (1.69) – 2.02	1.12 – (1.51) – 2.21	

The mantle pigment on the visceral coil of mature individuals of *A. subaquatilis* does not occur in A. cf. brazieri. The internal pigmentation of *A. subaquatilis* is generally lighter than that of A. cf. brazieri. The difference in the relative size of the bulbous termination of the praeputium is a consistent distinction in the male reproductive system of the two species, as are the shape of the spermatheca and the width of the spermathecal duct in the female system. Apart from the larger commissural lobule in A. cf. brazieri, mature *A. subaquatilis* formed a distinctly demarcated and ellipsoid lobe from each cerebral ganglion situated opposite the commissural lobule (namely adjacent to the buccal mass).

## ﻿Discussion

This study used an integrative approach, combining morphological data, mitogenome and nuclear rRNA gene cluster comparisons and phylogenetic analyses to assess the relationship between *A. subaquatilis* and A. cf. brazieri. Based on morphological features, these two snail taxa could be readily distinguished. The complete mitochondrial and nuclear rRNA gene clusters sequenced and compared herein are some of the first such data sets for the family Lymnaeidae and are among the very few available for the order Hygrophila (McQuirk et al. 2025). Analysis of mitochondrial and nuclear markers showed no significant difference in gene order or structure, although sufficient phylogenetically informative sites were reported to suggest that their divergence corresponded to species-level differentiation, and thus supported our morphological findings.

The patterns of nucleotide divergence in mitochondrial genes and the nuclear rRNA clusters of *A. subaquatilis* and A. cf. brazieri are distinct. Most mitochondrial genes exhibit low pairwise nucleotide divergence between the two species. For instance, the nucleotide p-distance for the *cox*1 gene is 2.3%, a value that was at the threshold between intra- and interspecific variation in other lymnaeid taxonomic studies (Bolotov et al. 2014; Vinarski et al. 2016, 2022; Aksenova et al. 2017, 2024; Lounnas et al. 2018; Ferreira et al. 2021; Falniowski et al. 2023), and is comparable to the minimal genetic divergence (p-distance = 2.4%) observed between *Ladislavella tumrokensis* and *L. elodes*, and between *Galba cubensis* and *G. neotropica* (see Vinarski et al. 2016; Ferreira et al. 2021). Moreover, the mitochondrial *16S*rRNA gene shows the lowest p-distance (0.6%) among all mitochondrial genes between the two species, further supporting previous findings that mitochondrial *16S*rRNA alone lacked sufficient phylogenetic resolution to delimit Australian *Austropeplea* species (Puslednik et al. 2009; Sukee et al. 2024). In contrast, although the sequences of the three nuclear rRNA genes (*18S*, *5.8S*, *28S*) of the two species are nearly identical, the observed differences in ITS regions (p-distance = 13.8% for ITS1; p-distance = 8.2% for ITS2) fall within the range of interspecific divergence commonly used in current lymnaeid taxonomic practices (see Vinarski et al. 2016; Ferreira et al. 2021; Aksenova et al. 2024). The higher divergence in ITS regions compared with mitochondrial genes may suggest the presence of introgression and incomplete lineage sorting within Australian *Austropeplea* (see Davis and Nixon 1992; Doyle 1992; Harrison and Larson 2014). Laboratory experiments showed that mating between species of *Austropeplea* was possible (Boray 1964b), although the fertility of hybrid offspring had not been demonstrated.

Despite the limitations of using *16S* gene and ITS2 markers alone for this group, we proceeded to use only these regions to make a phylogenetic comparison with data available for *Austropeplea*. The topology of the *16S* + ITS2 phylogenetic trees in this study and that of Puslednik et al. (2009) were largely consistent.

The newly sequenced *A. subaquatilis* and A. cf. brazieri were placed in two geographically structured lineages within *Austropeplea* (southern versus eastern Australia), consistent with regional structuring rather than constituting definitive evidence of species level divergence. Nevertheless, the limitations of the current markers were evident, as the main Australian *Austropeplea* lineage and several within group relationships showed polytomies, indicating unresolved relationships. These unresolved nodes may have reflected limited phylogenetic signal, rapid radiation, or incomplete lineage sorting, and thus warrant further investigation using additional genetic markers. Future studies employing complete mitochondrial genome sequences and nuclear rRNA gene clusters are warranted to address the unresolved relationships within the genus.

The morphological features of *A. subaquatilis* and A. cf. brazieri generally corresponded to the type B and type A morphs, respectively, as described by Boray and McMichael (1961). Morphologically, the differences between the two species were primarily focused on the shell, mantle extension, nervous system, and the reproductive system. The differences in the reproductive system between *A. subaquatilis* and A. cf. brazieri were distinct. The morphological difference in the bulbous termination of the praeputium may suggested underlying internal structural differences, which warrant further investigation in future studies. The nervous systems of *A. subaquatilis* and A. cf. brazieri differed, particularly in the size of the commissural lobule and the presence of additional lobes arising from the cerebral ganglia in *A. subaquatilis*. These differences were not reported in previous studies. The lack of comparative neuroanatomical studies on this group currently limited our ability to assess whether these features were structurally significant or how they related to other members of the Lymnaeidae. Further research is needed to clarify their nature and taxonomic relevance. The two unique features mentioned by Puslednik et al. (2009) for the South Australian samples (*A. subaquatilis* in this study), namely the longer cephalic tentacles (twice as long as wide) and the prostate (tube) being much longer than the female reproductive system, were also observed in this study. However, the recognition of these traits is largely depended on how much contraction had occurred due to preservation, as these features were less pronounced in contracted material compared to the other distinguishing features noted above.

The external features of the head-foot and mantle of *A. subaquatilis* included a broad foot with the shell enveloped by the mantle. In its natural habitat, *A. subaquatilis* was observed living in extremely dense water milfoil (*Myriophyllum* spp.) environments, which presumably necessitated moving between tightly packed branches. The reduction of shell-related hindrance may have enhanced the animal’s ability to survive in such environments. The abnormally high mucus production in *A. subaquatilis* when stimulated may have served as an alternative defence mechanism against predators, perhaps compensating for the fragile shell. This trait was commonly observed in land slugs and semi-slugs (Luchtel and Deyrup-Olsen 2001). The mantle extension may also have functioned to enhance cutaneous respiration, thereby reducing the frequency of surfacing for air or adapting to hypoxic conditions caused by intensified respiration of aquatic plants at night (Russell-Hunter 1978). The degree of mantle extension has not been well characterised across species in this family, with only the European *Myxas glutinosa* (O. F. Müller, 1774) explicitly documented as having a fully shell-enveloping mantle, similar to what was observed in *A. subaquatilis* (Hubendick 1951; Stadnichenko 2004). However, based on the phylogenetic relationships of *Austropeplea* from Tasmania and South Australia reconstructed in this study and previous research (Puslednik et al. 2009), it appeared that the development of mantle extensions in *Austropeplea* was not an isolated occurrence. Boray and McMichael (1961) noted that mantle extension in their type B morphs tended to diminish or disappear over successive laboratory generations. This observation closely resembled the dwarf forms we observed. However, these dwarf individuals regained mantle extension once placed in favourable environmental conditions, in contrast to A. cf. brazieri, in which this trait appeared to be permanently absent. Further studies are required to clarify the mechanism of mantle extension and to determine its biological or ecological relevance.

Taken together, the integrative evidence presented herein supported species-level divergence between *A. subaquatilis* and A. cf. brazieri. While mitochondrial divergence alone approached the threshold of interspecific separation, the pronounced differences in ITS regions, along with the morphological traits, provided a coherent framework for species delimitation. Nevertheless, the phylogeny of this group remains poorly resolved. Continued efforts involving additional phylogenetically informative genetic datasets and comparative anatomical studies will be essential for resolving outstanding taxonomic uncertainties and understanding the evolutionary dynamics of this group.

## Supplementary Material

XML Treatment for Austropeplea subaquatilis

XML Treatment for Austropeplea cf. brazieri

## ﻿Additional information

### Use of AI

No use of AI was reported.

### Author ORCIDs

Zhe-Yu Chen https://orcid.org/0000-0002-4150-8906

Tanapan Sukee https://orcid.org/0000-0003-3181-5045

Anson V. Koehler https://orcid.org/0000-0001-8330-6416

Bonnie L. Webster https://orcid.org/0000-0003-0930-9314

Robin B. Gasser https://orcid.org/0000-0002-4423-1690

Winston F. Ponder https://orcid.org/0000-0002-8600-3952

Neil D. Young https://orcid.org/0000-0001-8756-229X

### Data availability

All of the data that support the findings of this study are available in the main text or Supplementary Information.

## Supplementary materials

Supplementary material 1 Location, code, and NCBI GenBank accession numbers of the lymnaeid snail nucleotide sequences used in this study Data type pdf https://binary.pensoft.net/file/1434606This dataset is made available under the Open Database License (http://opendatacommons.org/licenses/odbl/1.0/). The Open Database License (ODbL) is a license agreement intended to allow users to freely share, modify, and use this Dataset while maintaining this same freedom for others, provided that the original source and author(s) are credited.Zhe-Yu Chen, Tanapan Sukee, Anson V. Koehler, Bonnie L. Webster, Robin B. Gasser, Winston F. Ponder, Neil D. Young

## References

[B1] Aksenova O Vinarski M Bolotov I Kondakov A Bespalaya Y Tomilova A Paltser I Gofarov M (2017) Two *Radix* spp. (Gastropoda: Lymnaeidae) endemic to thermal springs around Lake Baikal represent ecotypes of the widespread *Radix auricularia*. Journal of Zoological Systematics and Evolutionary Research 55(4): 298–309. 10.1111/jzs.12174

[B2] Aksenova OV Vinarski MV Itagaki T Ohari Y Oshida T Kim SK Lee JH Kondakov AV Khrebtova IS Soboleva AA Travina OV Sokolova SE Palatov DM Bespalaya YV Vikhrev IV Gofarov MY Bolotov IN (2024) Taxonomy and trans-Beringian biogeography of the pond snails (Gastropoda: Lymnaeidae) of East Asia: an integrative view. Zoological Journal of the Linnean Society 201(4): zlae083. 10.1093/zoolinnean/zlae083

[B3] Bernt M Donath A Jühling F Externbrink F Florentz C Fritzsch G Pütz J Middendorf M Stadler PF (2013) MITOS: Improved *de novo* metazoan mitochondrial genome annotation. Molecular Phylogenetics and Evolution 69(2): 313–319. 10.1016/j.ympev.2012.08.023 22982435

[B4] Bolotov I Bespalaya Y Aksenova O Aksenov A Bolotov N Gofarov M Kondakov A Paltser I Vikhrev I (2014) A taxonomic revision of two local endemic *Radix* spp. (Gastropoda: Lymnaeidae) from Khodutka geothermal area, Kamchatka, Russian Far East. Zootaxa 3869(5): 585–593. 10.11646/zootaxa.3869.5.9 25283942

[B5] Boray JC (1964a) Studies on the ecology of *Lymnaea tomentosa*, the intermediate host of *Fasciola hepatica*. 1. History, geographical distribution, and environment. Australian Journal of Zoology 12(2): 217–230. 10.1071/ZO9640217

[B6] Boray JC (1964b) Studies on the ecology of *Lymnaea tomentosa*, the intermediate host of *Fasciola hepatica*. 2. The sexual behaviour of *Lymnaea tomentos*a. Australian Journal of Zoology 12(2): 231–238. 10.1071/ZO9640231

[B7] Boray JC (1969) Experimental Fascioliasis in Australia. In: Dawes B (Ed.) Advances in Parasitology.Academic Press, 95–210. 10.1016/S0065-308X(08)60435-2 4935272

[B8] Boray JC (1978) The potential impact of exotic *Lymnaea* spp. on fascioliasis in Australasia. Veterinary Parasitology 4(2): 127–141. 10.1016/0304-4017(78)90004-3

[B9] Boray JC McMichael DF (1961) The identity of the Australian lymnaeid snail host of *Fasciola hepatica* L. and its response to environment. Marine and Freshwater Research 12(2): 150–163. 10.1071/MF9610150

[B10] Cotton BC (1942) Some Australian freshwater Gastropoda. Transactions of the Royal Society of South Australia 66: 75–82.

[B11] Cotton BC Godfrey FK (1938) A systematic list of the Gastropoda, the marine freshwater and land univalve Mollusca of South and Central Australia. Malacological Society of South Australia Publication No. 1, 44 pp.

[B12] Davis JI Nixon KC (1992) Populations, genetic variation, and the delimitation of phylogenetic species. Systematic Biology 41(4): 421–435. 10.1093/sysbio/41.4.421

[B13] Doyle JJ (1992) Gene trees and species trees: Molecular systematics as one-character taxonomy. Systematic Botany 17(1): 144–163. 10.2307/2419070

[B14] Falniowski A Jaszczyńska A Hofman S (2023) *Radix rufescens* (J. E. Gray, 1822) (Gastropoda: Lymnaeidae), a new species for Oman and Arabian Peninsula. Acta Zoologica Academiae Scientiarum Hungaricae 69(3): 303–312. 10.17109/AZH.69.3.303.2023

[B15] Ferreira APPN Costa ALO Becattini RM Ferreira MAND da Paixão HPR Coscarelli D Vidigal THDA Lima W dos S Pereira CA de J (2021) Integrative taxonomy: Combining molecular and morphological characteristics to identify Lymnaea (Galba) cubensis, intermediate host of *Fasciola hepatica.* Revista Brasileira de Parasitologia Veterinária = Brazilian Journal of Veterinary Parasitology : Órgão Oficial do Colégio Brasileiro de Parasitologia Veterinária 30(2): e026320. 10.1590/s1984-29612021052 34161492

[B16] Fourdrilis S de Frias Martins AM Backeljau T (2018) Relation between mitochondrial DNA hyperdiversity, mutation rate and mitochondrial genome evolution in *Melarhaphe neritoides* (Gastropoda: Littorinidae) and other Caenogastropoda. Scientific Reports 8(1): 17964. 10.1038/s41598-018-36428-7 30568252 PMC6299273

[B17] Ghiselli F Gomes-dos-Santos A Adema CM Lopes-Lima M Sharbrough J Boore JL (2021) Molluscan mitochondrial genomes break the rules. Philosophical Transactions of the Royal Society of London. Series B, Biological Sciences 376(1825): 20200159. 10.1098/rstb.2020.0159 PMC8059616 33813887

[B18] Grant JR Enns E Marinier E Mandal A Herman EK Chen C Graham M Van Domselaar G Stothard P (2023) Proksee: In-depth characterization and visualization of bacterial genomes. Nucleic Acids Research 51(W1): W484–W492. 10.1093/nar/gkad326 PMC10320063 37140037

[B19] Harrison RG Larson EL (2014) Hybridization, introgression, and the nature of species boundaries. The Journal of Heredity 105: 795–809. 10.1093/jhered/esu033 25149255

[B20] Hubendick B (1951) Recent Lymnaeidae: Their variation, morphology, taxonomy, nomenclature and distribution. Kungliga Svenska Vetenskapsakademiens Handlingar. Fjärde Serien 3: 1–223.

[B21] Iredale T (1943) A basic list of the freshwater Mollusca of Australia. Australian Zoologist 10(2): 188–230.

[B22] Iredale T (1944) Guide to the freshwater shells of New South Wales. Pt II. Australian Naturalist 11: 113–127.

[B23] Kalyaanamoorthy S Minh BQ Wong TKF von Haeseler A Jermiin LS (2017) ModelFinder: Fast model selection for accurate phylogenetic estimates. Nature Methods 14(6): 587–589. 10.1038/nmeth.4285 28481363 PMC5453245

[B24] Liu G-H Wang S-Y Huang W-Y Zhao G-H Wei S-J Song H-Q Xu M-J Lin R-Q Zhou D-H Zhu X-Q (2012) The complete mitochondrial genome of *Galba pervia* (Gastropoda: Mollusca), an intermediate host snail of *Fasciola* spp. PLoS ONE 7(7): e42172. 10.1371/journal.pone.0042172 PMC3406003 22844544

[B25] Lounnas M Correa AC Alda P David P Dubois M-P Calvopiña M Caron Y Celi-Erazo M Dung BT Jarne P Loker ES Noya O Rodríguez-Hidalgo R Toty C Uribe N Pointier J-P Hurtrez-Boussès S (2018) Population structure and genetic diversity in the invasive freshwater snail *Galba schirazensis* (Lymnaeidae). Canadian Journal of Zoology 96(5): 425–435. 10.1139/cjz-2016-0319

[B26] Luchtel DL Deyrup-Olsen I (2001) Body wall: form and function. In: Barker GM (Ed.) The biology of terrestrial molluscs.CABI Publishing, Oxon, 147–178. 10.1079/9780851993188.0147

[B27] McQuirk KA DeCore JM Castillo MG Adema CM (2025) Rewilding shows differential fitness of sympatric *Physella acuta* (Draparnaud, 1805) snail lineages. Aquatic Ecology 59(2): 435–454. 10.1007/s10452-025-10172-3

[B28] Minh BQ Nguyen MAT von Haeseler A (2013) Ultrafast approximation for phylogenetic bootstrap. Molecular Biology and Evolution 30(5): 1188–1195. 10.1093/molbev/mst024 23418397 PMC3670741

[B29] Müller OF (1774) Vermium terrestrium et fluviatilium, seu animalium infusorium, Helminthicorum, et testaceorum, non marinorum, succincta historia. vol 2. Havniae et Lipsiae, apud Heineck et Faber, ex officina Mölleriana. I-XXXVI, 1–214 pp. [10 unnumbered pages] 10.5962/bhl.title.46299

[B30] Pfeifer B Wittelsbürger U Ramos-Onsins SE Lercher MJ (2014) PopGenome: An efficient Swiss Army knife for population genomic analyses in R. Molecular Biology and Evolution 31(7): 1929–1936. 10.1093/molbev/msu136 24739305 PMC4069620

[B31] Pfeiffer L (1855) Descriptions of fifty-seven new species of Helicea, from Mr Cunning’s collection. Proceedings of the Zoological Society of London 1854: 286–298. 10.1111/j.1469-7998.1854.tb07277.x

[B32] Ponder WF Waterhouse JH (1997) A new genus and species of Lymnaeidae from the lower Franklin River, south western Tasmania, Australia. The Journal of Molluscan Studies 63(3): 441–468. 10.1093/mollus/63.3.441

[B33] Ponder WF Hallan A Shea ME Clark SA Richards K Klunzinger MW Kessner V (2024) Australian Freshwater Molluscs. Revision 2A. https://keys.lucidcentral.org/keys/v3/freshwater_molluscs/

[B34] Puslednik L Ponder WF Dowton M Davis AR (2009) Examining the phylogeny of the Australasian Lymnaeidae (Heterobranchia: Pulmonata: Gastropoda) using mitochondrial, nuclear and morphological markers. Molecular Phylogenetics and Evolution 52(3): 643–659. 10.1016/j.ympev.2009.03.033 19362157

[B35] Rafinesque CS (1815) Analyse de la nature ou Tableau de l’univers et des corps organisés. Published by the author, Palermo, 224 pp. 10.5962/bhl.title.106607

[B36] Rambaut A Drummond AJ Xie D Baele G Suchard MA (2018) Posterior Summarization in Bayesian phylogenetics using Tracer 1.7. Systematic Biology 67(5): 901–904. 10.1093/sysbio/syy032 29718447 PMC6101584

[B37] Ronquist F Teslenko M van der Mark P Ayres DL Darling A Höhna S Larget B Liu L Suchard MA Huelsenbeck JP (2012) MrBayes 3.2: Efficient Bayesian phylogenetic inference and model choice across a large model space. Systematic Biology 61(3): 539–542. 10.1093/sysbio/sys029 22357727 PMC3329765

[B38] Russell-Hunter WD (1978) Ecology of freshwater pulmonates. In: Fretter V Peake J (Eds) Pulmonates, vol 2A.Academic Press, London, 335–384.

[B39] Smith EA (1882) On the freshwater shells of Australia. Journal of the Linnean Society of London, Zoology 16(92): 255–316. 10.1111/j.1096-3642.1882.tb02283.x

[B40] Stadnichenko AP (2004) Pond snails and limpet snails (Lymnaeidae and Acroloxidae) of Ukraine. Tsentr uchebnoi literatury, Kiev, 327 pp. [in Russian]

[B41] Sukee T Koehler AV Webster BL Gauci CG Fogarty CE Ponder WF Gasser RB Young ND (2024) Mitochondrial genome of the fluke pond snail, Austropeplea cf. brazieri (Gastropoda: Lymnaeidae). Parasites & Vectors 17(1): 283. 10.1186/s13071-024-06358-7 38956636 PMC11218368

[B42] Suwancharoen C Phuangsri C Siriwechviriya P Bunsong T Japa O (2023) Diversity of trematode cercariae among naturally infected lymnaeid snails from Phayao, Thailand. Parasitology Research 122(11): 2691–2708. 10.1007/s00436-023-07971-8 37698606

[B43] Tate R (1880) Descriptions of some new species of south Australian Pulmonifera. Transactions of the Royal Society of South Australia 3: 102–104.

[B44] Tenison Woods JE (1876) On the freshwater shells of Tasmania. Papers and Proceedings of the Royal Society of Tasmania 1875: 66–82. 10.5962/bhl.part.9781

[B45] Vázquez AA Alba A Alda P Vittecoq M Chapuis E Faugère D Pointier J-P Hurtrez-Boussès S (2023) Lymnaeid snails and the transmission of Fasciolosis: Understanding the differential risks from local to global scale. In: Vinarski MV Vázquez AA (Eds) The Lymnaeidae: A Handbook on Their Natural History and Parasitological Significance.Springer International Publishing, Cham, 359–394. 10.1007/978-3-031-30292-3_13

[B46] Vinarski MV Pointier J-P (2023) Conchological and anatomical identification of the lymnaeid snails. In: Vinarski MV Vázquez AA (Eds) The Lymnaeidae: A Handbook on Their Natural History and Parasitological Significance.Springer International Publishing, Cham, 103–120. 10.1007/978-3-031-30292-3_4

[B47] Vinarski M Aksenova O Bespalaya Y Bolotov I Gofarov M Kondakov A (2016) Ladislavella tumrokensis: The first molecular evidence of a Nearctic clade of lymnaeid snails inhabiting Eurasia. Systematics and Biodiversity 14(3): 276–287. 10.1080/14772000.2016.1140244

[B48] Vinarski MV Clewing C Albrecht C (2019) Lymnaeidae Rafinesque, 1815. In: Lydeard C Cummings S (Eds) Freshwater Mollusks of the World.Johns Hopkins University Press, Baltimore, 158–162.

[B49] Vinarski MV Von Oheimb PV Aksenova OV Gofarov MY Kondakov AV Nekhaev IO Bolotov IN (2022) Trapped on the roof of the world: taxonomic diversity and evolutionary patterns of Tibetan Plateau endemic freshwater snails (Gastropoda: Lymnaeidae: Tibetoradix). Integrative Zoology 17(5): 825–848. 10.1111/1749-4877.12600 34750963

[B50] Vinarski MV Aksenova OV Bolotov IN Vázquez AA Alda P Pointier J-P Hurtrez-Boussès S (2023) Biogeography of the living Lymnaeidae. In: Vinarski MV Vázquez AA (Eds) The Lymnaeidae: A Handbook on Their Natural History and Parasitological Significance.Springer International Publishing, Cham, 183–206. 10.1007/978-3-031-30292-3_7

[B51] Wang M Kong L (2019) pblat: A multithread blat algorithm speeding up aligning sequences to genomes. BMC Bioinformatics 20(1): 28. 10.1186/s12859-019-2597-8 30646844 PMC6334396

[B52] Wickham H (2016) ggplot2. Elegant Graphics for Data Analysis. Second Edition. Springer International Publishing, Cham, 260 pp. 10.1007/978-3-319-24277-4

