# Supplementary material for: ﻿Mitogenome and nuclear rRNA gene cluster of Austropeplea subaquatilis (Tate, 1880) from South Australia, with molecular and morphological comparison of A. cf. brazieri (Smith, 1882) from Victoria (Gastropoda, Hygrophila, Lymnaeidae)
Source: Zookeys. 2025 Oct 9;1255:41–62. doi: 10.3897/zookeys.1255.164109 (PMC12531821; doi:10.3897/zookeys.1255.164109)
Supplement: Supplementary material 1 — Location, code, and NCBI GenBank accession numbers of the lymnaeid snail nucleotide sequences used in this study [file zookeys-1255-041_article-164109__-s001.pdf]

Supplementary Table 1. Location, code, and NCBI GenBank accession numbers of the lymnaeid snail nucleotide sequences used in this study.

Species	Code	Locality	*16S*	ITS2	References
Austropeplea cf. *brazieri*	EA-1	Guyra, New South Wales, Australia (30°13.330'S, 151°40.170'E)	EU556225	EU556270	Puslednik et al. (2009)
Austropeplea cf. *brazieri*	EA-2	Walcha, New South Wales, Australia (30°54.586'S, 151°17.306'E)	EU556226	EU556271	Puslednik et al. (2009)
Austropeplea cf. *brazieri*	EA-3	Penrith, New South Wales, Australia (33°38.500'S, 150°41.500'E)	EU556227	EU556272	Puslednik et al. (2009)
Austropeplea cf. *brazieri*	EA-4	Windsor, New South Wales, Australia (33°37.000'S, 150°49.000'E)	EU556228	EU556273	Puslednik et al. (2009)
Austropeplea cf. *brazieri*	EA-5	Braidwood, New South Wales, Australia	EU556229	EU556274	Puslednik et al. (2009)

		(35°31.483'S, 149°31.700'E)			
Austropeplea cf. *brazieri*	EA-6A	Kosciuszko Plateau, New South Wales, Australia (36°24.368'S, 148°19.064'E)	EU556230	EU556275	Puslednik et al. (2009)
Austropeplea cf. *brazieri*	EA-6B	Kosciuszko Plateau, New South Wales, Australia (36°24.368'S, 148°19.064'E)	EU556231	EU556276	Puslednik et al. (2009)
Austropeplea cf. *brazieri*	EA-7	Bombala, New South Wales, Australia (37°08.783'S, 149°28.087'E)	EU556232	EU556277	Puslednik et al. (2009)
Austropeplea cf. *brazieri*	EA-8	Bemboka, New South Wales, Australia (36°34.500'S, 149°41.467'E)	EU556233	EU556278	Puslednik et al. (2009)
Austropeplea cf. *brazieri*	EA-9A	Castlemaine, Victoria, Australia	EU556248	EU556293	Puslednik et al. (2009)

		(37°19.277'S, 144°21.777'E)			
Austropeplea cf. *brazieri*	EA-9B	Castlemaine, Victoria, Australia (37°19.277'S, 144°21.777'E)	EU556249	EU556294	Puslednik et al. (2009)
Austropeplea cf. *brazieri*	AUS1	Werribee South, Victoria, Australia (37°56.682’S, 144°41.931’E)	PP100270	PV593740	Sukee et al. (2024) and this study
Austropeplea tomentosa	NZn-1	East Cape, North Island, New Zealand (37°39.400'S, 178°29.610'E)	EU556234	EU556279	Puslednik et al. (2009)
Austropeplea tomentosa	NZn-2	North of Napier, North Island, New Zealand (39°13.040'S, 176°53.380'E)	EU556235	EU556280	Puslednik et al. (2009)
Austropeplea tomentosa	NZs-1	Arthur’s Pass, South Island, New Zealand (42°54.340'S, 171°33.618'E)	EU556236	EU556281	Puslednik et al. (2009)

Austropeplea tomentosa	NZs-2	Avon River, Christchurch, New Zealand (43°32.000'S, 172°38.000'E)	EU556237	EU556282	Puslednik et al. (2009)
Austropeplea tomentosa	NZs-3	Little River, South Island, New Zealand (43°44.929'S, 172°49.450'E)	EU556238	EU556283	Puslednik et al. (2009)
Austropeplea tomentosa	NZs-4	Mole Lake, South Island, New Zealand (45°00.437'S, 168°34.384'E)	EU556239	EU556284	Puslednik et al. (2009)
Austropeplea subaquatilis	SA-1	Penola, South Australia, Australia (37°15.299'S, 140°26.114'E)	EU556240	EU556285	Puslednik et al. (2009)
Austropeplea subaquatilis	SA-2	Mt Gambier, South Australia, Australia (37°22.301'S, 140°12.624'E)	EU556241	EU556286	Puslednik et al. (2009)

Austropeplea subaquatilis	SA-3	Millicent, South Australia, Australia (36°04.688'S, 140°06.075'E)	EU556242	EU556287	Puslednik et al. (2009)
Austropeplea subaquatilis	AB291	Thornlea, South Australia, Australia (37°22.135’S, 140°12.313’E)	PV749633	PV593739	This study
Austropeplea huonensis	TAS-1	Launceston, Tasmania, Australia (41°26.875'S, 147°07.286'E)	EU556243	EU556288	Puslednik et al. (2009)
Austropeplea huonensis	TAS-2	South Esk River, Tasmania, Australia (42°16.800'S, 147°35.400'E)	EU556244	EU556289	Puslednik et al. (2009)
Austropeplea huonensis	TAS-3	Lake Augusta, Tasmania, Australia (41°52.340'S, 146°30.779'E)	EU556245	EU556290	Puslednik et al. (2009)
Austropeplea huonensis	TAS-4	Clyde River, Tasmania,	EU556246	EU556291	Puslednik et al. (2009)

		Australia (42°21.385'S, 147°01.395'E)			
Austropeplea huonensis	TAS-5	Lemont, Tasmania, Australia (42°16.807'S, 147°35.411'E)	EU556247	EU556292	Puslednik et al. (2009)
Austropeplea hispida	N/A	Franklin River, Tasmania, Australia (42°32.450'S, 145°45.202'E)	EU556268	EU556311	Puslednik et al. (2009)
Austropeplea hispida	N/A	Franklin River, Tasmania, Australia (42°32.450'S, 145°45.202'E)	EU556269	EU556312	Puslednik et al. (2009)
Orientogalba viridis	N/A	Asia	NC018536	OR030153	Liu et al. (2012) and Suwancharoen et al. (2023)
